# Does Miniscrew-Assisted Rapid Palatal Expansion Influence Upper Airway in Adult Patients? A Scoping Review

**DOI:** 10.3390/dj12030060

**Published:** 2024-03-01

**Authors:** Mariachiara Benetti, Luca Montresor, Daniele Cantarella, Nicoletta Zerman, Enrico Spinas

**Affiliations:** 1 Department of Surgical Science, Post Graduate School of Orthodontics, University of Cagliari, Via Ospedale, 01924 Cagliari, Italy; mariachiara.benetti@gmail.com; 2 Department of Biomedical, Surgical and Dental Sciences, University of Milan, Via Commenda 10, 20122 Milan, Italy; danielecant@hotmail.com; 3 Department of Pediatric Dentistry and Dental Hygiene, University of Verona, Via San Marco 121, 37138 Verona, Italy; nicoletta.zerman@univr.it

**Keywords:** airway, adult, maxillary expansion, miniscrew, MSE, CBCT, scoping review

## Abstract

(1) Objective: This scoping review evaluates the effects of miniscrew-assisted rapid palatal expansion (MARPE) on different regions of the upper airway in adult patients and investigates various methods of measurement. (2) Methods: The search encompassed Pubmed, Cochrane Library, Scopus and Web of Science. This review was conducted following the PRISMA_ScR guidelines, and the inclusion criteria for examined studies were chosen in accordance with the PICOS framework. (3) Results: Seven studies were included in this review, comprising four retrospective studies, one prospective and two case reports. All studies involved the use of Cone Beam Computed Tomography (CBCT) for measurements of the areas of interest. The percentage of increase in the volume of the nasal cavity varied between 31% and 9.9%, depending on the study. Volumetric variations in the nasopharynx were reported as increases between T0 (before expansion) and T1 (immediately after expansion) of 6.4%, 20.7% and 14.1%. All studies considered T0 before expansion and T1 immediately after expansion. Only one study evaluated remote follow-up to assess if the results were maintained after one year. (4) Conclusions: MARPE appears to lead to a statistically significant increase in the upper airway, especially in the nasal cavity and nasopharynx immediately after expansion. However, further prospective and retrospective trails with long-term controls are required to verify the effects of MARPE on the upper airway.

## 1. Introduction

Miniscrew-assisted rapid palatal expansion (MARPE) is a procedure employed to address maxillary transverse discrepancies in adult patients. Nevertheless, the impacts of this expansion technique on the upper airway have not yet been fully elucidated [1]. 

The contraction of the upper jaw is a relatively common malocclusion, observed in both young and adult patients [1], with a prevalence ranging between 10% and 23% [2,3]. This malocclusion is often characterized by a narrow upper jaw, along with unilateral or bilateral crossbite, dental crowding and a high palatal vault. In comparison to individuals with normal occlusions, these patients often experience a reduction in upper airway (UA) volume [4]. Constriction of the airways results in a decreased airflow to the lungs, thereby negatively impacting an individual’s growth, development and overall health [5].

Several studies have reported a correlation between a transverse deficit in the maxilla and an increased incidence of obstructive sleep apnea syndrome (OSAS) [6,7].

The role of orthodontists and pediatric dentists in intercepting OSAS is essential to correct orthodontic alterations that may favor the development of this condition. Orthodontic treatment seems to reduce the severity of OSAS by increasing the airspace and improving airflow through orthopedic expansion of the upper jaw and mandibular advancement. Rapid palatal expanders and MARPE could be useful in the treatment of OSAS [8].

Addressing this issue necessitates a skeletal expansion of the upper jaw, aimed at widening the maxilla itself, rather than merely expanding the dental arches by moving the teeth in the dentoalveolar processes. The most commonly employed device to achieve this orthopedic effect in growing numbers of patients is the Rapid Palate Expander (RPE), which can separate the median palatine suture that is not yet fully ossified during developmental stages. However, in adult patients, the median palatine suture is either partially or completely ossified, rendering the RPE less suitable as the treatment of choice. This device would primarily yield dental effects rather than the desired orthopedic effect.

Historically, for treating adult patients affected by upper jaw contraction, the preferred approach has been surgically assisted rapid palatal expansion (SARPE). This technique involves the execution of osteotomies aimed at reducing the resistance to expansion by creating an aperture through which the median suture can be separated. However, it is important to note that this procedure carries an increased risk of complications and imposes high biological costs [9]. The literature reports several complications related to SARPE, including significant hemorrhages, gingival recessions, root resorptions, infections, loss of vitality of dental elements, risks of nerve damage to the maxillary nerve, periodontal damage, sinusitis, flaring of the ala of the nose, extrusions of the teeth anchored to the device, non-symmetrical expansions as well as general anesthetic risks [10].

As an alternative to surgery, the MARPE technique has been proposed [11]. This technique has received attention from clinicians because it is less invasiveness than SARPE with fewer collateral effects when compared with classic RME. In this approach, orthodontic miniscrews positioned on the palatine vault serve as a skeletal anchorage for the rapid palatal expander, thereby reducing the adverse dento-alveolar effects associated with RPE and mitigating the biological costs associated with SARPE [12]. Studies have demonstrated that MARPE can serve as a valid alternative for treating the transverse deficit of the maxilla in late adolescence, yielding statistically significant changes in both intermolar and alveolar diameter [13]. Various types of expanders connected to palatal miniscrews have been proposed, categorizing them into two types: hybrid or bone borne [14]. Hybrid expanders offer both skeletal and dental support, while the bone-borne expanders anchor solely to the palatal vault, without contacting the teeth.

A specific type of MARPE is the Maxillary Skeletal Expander (MSE), designed to induce a more parallel expansion in the anteroposterior direction of the palatal suture compared to other devices [15,16,17]. This unique expansion directs forces posteriorly and superiorly into the nasal cavity by engaging both layers of palatal and nasal cortical bone. Recently, numerous studies have demonstrated volumetric changes in the upper airway after treatment with a traditional rapid palatal expander [18,19,20,21]. Moreover, MARPE seems to decrease total resistance in the upper airway and airflow pressure. However, it remains unclear whether and how MARPE treatments in adult patients, with minimal growth potential, can yield similar benefits.

## 2. Materials and Methods

This review adhered to the PRISMA_ScR (Preferred Reporting Items for Systematic reviews and Meta-Analyses—extension for Scoping Reviews) guidelines [22].

### 2.1. Research Questions 

The primary focus in this review was to elucidate the influence of miniscrew-assisted skeletal expansion (MARPE) treatments on the upper airways in adult patients. Specifically, the inquiry sought to understand how MARPE affects the upper airways and whether these effects are sustained post-retention.

### 2.2. Eligibility Criteria

Specifically, the inquiry aims to comprehend the impact of miniscrew-assisted skeletal expansion (MARPE) on the upper airways and ascertain the sustainability of these effects post-retention. 

**Figure 1:**
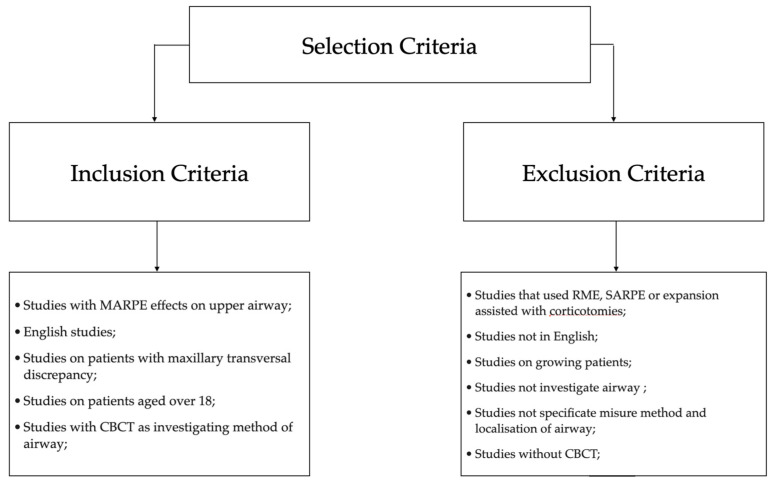
Diagram showing the inclusion and exclusion criteria used for this review.

The inclusion criteria for the examined studies were chosen in accordance with the PICOS (Population, Intervention, Comparison, Outcome, Study Desing) principles and are delineated in Figure 1.

(1) Population: Patients with a transverse skeletal discrepancy of maxilla treated with MARPE technique, specifically focusing on individual over 18 years of age. We opted to narrow the scope of the review to studies involving adult patients, aged 18 and above, to minimize the potential influence of physiological growth on outcomes, thereby enhancing the reliability of the results. It is crucial to note that, in growing numbers of patients, variations observed can be attributed to normal development processes, rather than the specific method used.
(2) Intervention: MARPE micro-implant-assisted rapid palatal expansion.
(3) Comparison: Comparing upper airway conditions before treatment (T0) to after treatment (T1) and assessing the sustainability of results over time (T2).
(4) Outcome: Considering volumetric changes or sectional areas on CBCT or CT scans performed before and after the expansion treatment.
(5) Study design: For inclusion, articles needed to have full English text, without restriction on the publication year. Accepted study designs encompassed observational studies, randomized clinical trials, case reports and case series.

### 2.3. Selection Criteria and Search Strategy

Articles were systematically searched across electronic databases, including Pubmed, Cochrane Library, Scopus and Web of Science. The search, conducted independently by two authors (M.B. and L.M.), spanned from June 2023 to October 2023, with no restriction on the publication year. Titles and abstracts of potentially eligible articles were rigorously assessed. In cases where the abstract lacked adequate information, or was unavailable, the full text of the article was obtained and scrutinized. The final selection of articles was collaboratively curated by authors (M.B. and L.M.). In instances of uncertainties or discrepancies, a third author (E.S.) provided input. 

For the search strategy, the following keywords were utilized: ((airway) AND (miniscrew OR microimplant assisted maxillary expansion)), ((airway) AND (MARPE OR microimplant assisted rapid palatal expansion)), ((airway AND maxillary expansion OR palatal expansion)), ((airway) AND (MSE)).

The inclusion criteria are summarized in Figure 1.

The exclusion criteria are defined as follows: studies that used RME and SARPE, studies on growing numbers of patients and without CBCT. 

### 2.4. Study Quality Assessment 

The data extraction was conducted independently by two authors (M.B. and L.M.). To assess the level of agreement between reviewers, Cohen’s Kappa coefficient (K = 0.9) was calculated, indicating almost total agreement.

### 2.5. Data Items and Collection 

Two reviewers (M.B. and L.M.) extracted the following information from the studies: first author and year of publication, study type, details on the examined sample (number of participants, age and gender of patients), type of expander used in the treatment and the expander activation protocol, airway evaluation method region of the airway investigated, software used for image reconstruction, results obtained (changes in volume and area of the investigated region), percentage change between T0 and T1, and follow-up (T0–T2). 

Anatomically, the airways were categorized into nasal cavities, nasopharynx, oropharynx and hypopharynx. The extracted data were organized into a table for the final analysis.

## 3. Results

### 3.1. Study Election

**Figure 2:**
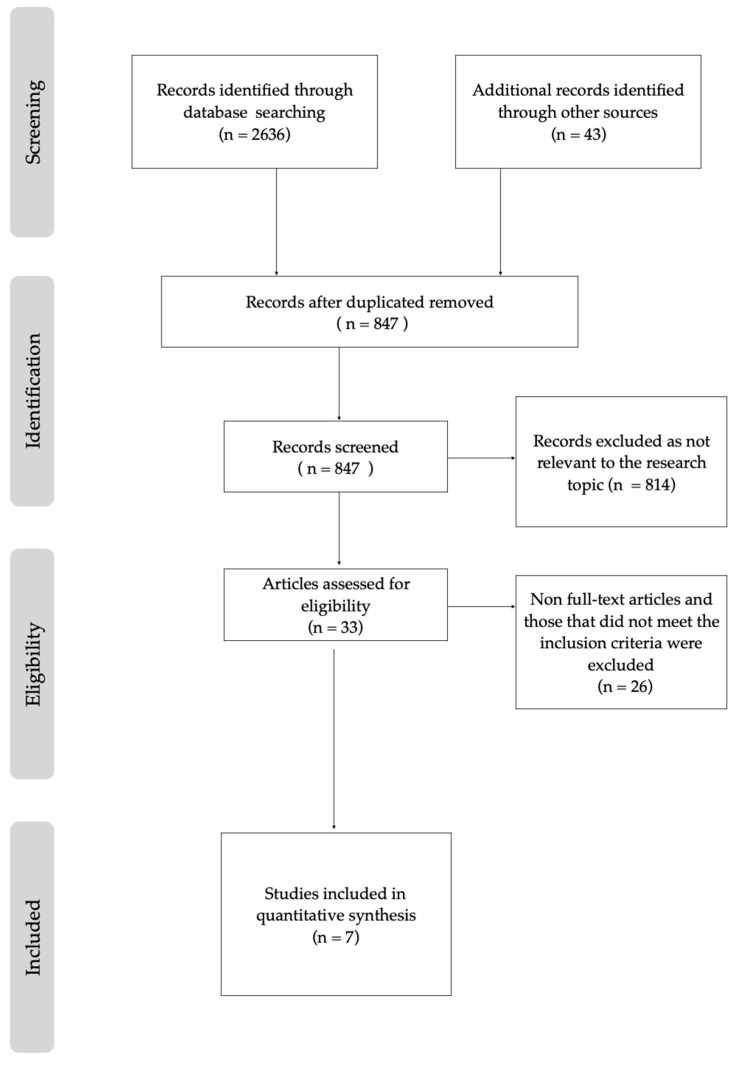
A flow diagram of the study selection process.

A total of 2636 publications were considered following a computer-assisted search. Following the removal of duplicates, 847 articles were initially identified. After scrutinizing the titles and abstracts, 33 studies were thoroughly examined. Of these, 26 articles were excluded for not fully meeting the inclusion criteria (6 due to not using CBCT/CT and 20 based on age restriction). Ultimately, only seven studies fully met the inclusion criteria and were included in this scoping review. All essential information from these studies is presented in a table for comprehensive analysis. A flow diagram of the study selection process is presented in Figure 2.

### 3.2. Study Design 

Within the selected studies, the distribution of study designs is as follows: four retrospective studies, one prospective study and two case reports. 

### 3.3. Type of Appliance and Activation Protocol 

Two studies [23,24] did not specify the type of device and simply mentioned treatment with MARPE. 

Three studies used MSE [25,26,27], while one employed a Hyrax device with hybrid anchoring (four bands and four miniscrews) [28], and another utilized a hybrid expander with four miniscrews [29]. The activation protocols varied; Li et al. and Garcez et al. [25,27] employed two activations per day until the discrepancy was resolved. Two other authors [26,28] utilized one activation per day until the discrepancy was corrected (range of 40–60 days; average 28 days). The activation protocols were not specified in the remaining studies [23,24,29].

### 3.4. Evaluation Method

All studies employed CBCT for measurements of the areas of interest, primarily utilizing volumetric measurements, However, one study compared area sections [24]. Additionally, Garcez et al. [27] incorporated respiratory tests (maximum inspiratory and expiratory pressure, peak expiratory oral flow, inspiratory nasal flow), Tang et al. [26] utilized computational fluid dynamics to describe the aerodynamic characteristics, Hur et al. [24] included computational fluid dynamics analysis and Shetty et al. [23] also performed linear measurements using CBCT. The characteristics of the included studies are summarized in Table 1.

### 3.5. Airway Aerea Localisation Evaluated

#### 3.5.1. Nasal Cavity

Four studies evaluated this anatomical region [24,25,27,28], while others did not. All studies detected an increase in the volume of the nasal cavities after expansion (T0–T1). The percentage of increase varied among the studies, ranging from 31% to 9.9%. Specifically, Garcez et al. [27] found an increase of 31%, Kim et al. [28] reported 9.9% and Li et al. [25] reported 16.2%.

A study by Hur et al. [24] noted more significant variation in the anterior portion of the nasal cavity compared to the posterior portion.

Notably, only one study [28] considered the difference between the start (T0) and post-retention (T2), reporting an increase in the volume of the nasal cavities one year after the end of expansion of 15.4%.

#### 3.5.2. Nasopharynx

Three studies [25,26,28] evaluated the volumetric variation in the nasopharynx, reporting increases between T0 and T1 of 6.4%, 20.7% and 14.1%, respectively.

Hur [24] observed a strong increasing trend in planes 1 and 2, ranging from 40.42% to 57.41% between T1 and T0.

#### 3.5.3. Oropharynx 

Tang et al. [26] evaluated the oropharynx, finding a T0–T1 volumetric increase of 8.84%.

Li et al. [25] examined the retropalatal and retrolingual areas, reporting T0–T1 increases of 5.7% and 11.26%, respectively. Additionally, an increase in the volume of the glossopharynx after retention (T2) was reported.

Hur et al. [24] reported an increase on the upper and middle parts of the oropharynx by 53.27% to 27.51% on planes 3 to 5. 

Anéris et al. [29] found no statistically significant volumetric differences between T0 and T1 in the constriction of the retrolingual and retropalatal regions; however, they observed a statistically significant total volumetric increase in the examined regions.

Shetty et al. [23] did not find statistically significant variations between oropharynx volumes in the retrolingual and retropalatal airways. 

#### 3.5.4. Hipopharynx

Only two studies [25,26] examined this anatomical region.

Li et al. [25] reported a decrease in hypopharyngeal volume between T0 and T1, but it was not statistically significant. Tang et al. [26] reported an increase of 8.84% between T0 and T1.

### 3.6. Follow-Up 

All studies considered T0 before expansion and T1 immediately after expansion, except Tang [26], who evaluated T1 three months after expansion, Hur et al. [24], who assessed T1 six months after expansion, and Anéris et al. [29] who conducted evaluations 120 days after expansion.

The only study that evaluated remote follow-up to assess whether the result was maintained was conducted by Kim et al. [28].

## 4. Discussion

The findings from this review strongly indicate that treatment with the MARPE technique influences the anatomy of the upper airways, with notable changes observed in the nasal cavities and nasopharynx. 

It is well established that developmental anomalies in the cranio-maxillofacial district affecting the upper airways represent a risk factor for the development of obstructive sleep apnea syndrome (OSAS). Specifically, studies have demonstrated that a transverse deficit of the upper jaw increases the risk of developing these issues [8,30,31]. Consequently, understanding how MARPE modifies the upper airways is crucial, especially in assessing its potential as a treatment for adult patients with both OSAS and transverse maxillary contractions. Presently, scientific evidence in this domain is limited and somewhat controversial. Further studies are warranted, with the focus on quantifying the reduction in incidence. Comparative analyses between subjects treated with MARPE, surgically assisted rapid palatal expansion (SARPE) and those not treated would contribute to a more comprehensive understanding of the efficacy of MARPE in addressing OSAS and maxillary transverse contraction in adults.

The median palatine suture has been described as a suture, with morphological characteristics that vary depending on the growth stages [32,33,34,35,36,37]. This underscores the importance of considering the growth and maturation stages of the skeletal grade when evaluating the outcome of expansion treatment. The variations observed could be influenced by or attributed to these variables.

In the examined literature, there is a notable absence of a reference parameter for subdividing samples based on these important factors. Currently, there are several methods for evaluating skeletal maturation, with the most commonly cited being the stage of maturation of the cervical vertebrae, the phalanx method or the degree of ossification in the median palatine suture [38]. The decision to restrict the examined sample to individuals over 18 years of age was made with an awareness of the potential disparity between chronological age and the actual stage of skeletal maturation.

In the studies analyzed, T1 was recorded at different times, albeit all with a very close T0–T1 time proximity. To enhance the reliability of these studies, establishing a uniform and more extended measurement time is essential. Additionally, a notable observation is the lack of examination in long-term stability in almost all cases. It is crucial to investigate whether these variations are sustained over time or if any relapse occurs.

The upper airway is a complex system of anatomical structures, comprising bones, cartilage and soft tissues. It plays a crucial role in functions, such as breathing, swallowing and phonation [39]. However objectively measuring the airways has always presented numerous challenges. In recent years, significant progress has been made in investigative techniques. While numerous measurement systems have been proposed to comprehend this intricate operating system, there is no singular approach. 

Indeed, the upper airway is a system with dynamic characteristics, leading to turbulent air flows, contractions or collapses at various levels, contingent on the patient’s health and needs. The optimal approach likely involves a combination of different techniques, encompassing physical, radiographic, endoscopic and acoustic methods [40].

Presently, cone beam computed tomography (CBCT) stands out as one of the most widely employed methods in studying the upper airway (UA) in orthodontic patients. However, a notable gap in the literature is the absence of standardized guidelines for comprehending UA morphology and, crucially, for defining its anatomical boundaries [41]. It is worth noting that image processing systems vary depending on the software used, potentially leading to divergent results.

The most relevant question about CBCT consists of the reliability of the methods of measurement. It has been advocated that it is not sufficient justification to support the use of CBCT by clinicians to assess a patient’s upper airway to diagnose OSA [42]. The reliability of upper airway assessment using CBCT has been investigated by several authors. It seems that reliability improves with examiner experience, and better results have been found in the oropharyngeal volume [43].

The most frequently examined anatomical region is the nasal cavity. In all the studies selected in our review that investigated this region [24,25,27,28], there is a consistent observation of an increase in this area following MARPE.

The nasopharynx emerges as the second-most-frequently cited area in the selected studies [25,26,28]. In each of these studies, all authors consistently report a volumetric increase in this region following expansion with MARPE.

The oropharynx comprises the glossopharynx and the palatopharynx. Three authors [25,26,29] identify volumetric increases in these regions, while only one author [23] reports no differences. It is important to bear in mind that many structures within the oropharynx, including the tongue, the hyoid bone and the soft palate, are mobile structures [13]. Their evaluation can be influenced and altered by factors, such as posture, gravity or movements associated with breathing or swallowing, that may inadvertently occur at the moment of image acquisition [1].

The hypopharynx was the subject of examination in only two articles: Li et al. [25] reported no statistically significant differences, while Tang et al. [26] observed an increase of 8.84%.

Research has demonstrated that posture influences the size of the upper airway (UA) [44,45]. In the supine position, the airways tend to become smaller, resulting in increased resistance [46]. Hence, it would be desirable to compare studies that conduct examinations in the same position. It is worth noting that the inclination of the head also influences the volume of the airways. Certain authors [47,48] examined variations in teleradiographs by tilting the head relative to the natural head position (NHP). Notably, inclinations of 20 degrees with respect to this reference point led to variations of almost 4 mm in the pharyngeal air space.

The vertical and sagittal facial pattern can also influence variations in UA size [42]. Modifying the maxillary transversality can lead to a repositioning of the mandible, and this can be influenced by the initial facial typology.

From a clinical perspective, it would be valuable to ascertain whether these variations have practical significance, particularly in terms of enhancing the patient’s quality of life and respiratory efficiency. To assess improvements in quality of life, comparison questionnaires such as the “Epworth Sleepiness Scale” or “Quebec Sleep Questionnaire” can be utilized before and after treatment. For the examination of respiratory effectiveness, respiratory tests, like “maximum inspiratory pressure (MIP)”, “maximum expiratory pressure (MEP)”, “oral expiratory peak flow” and “inspiratory nasal flow”, can be employed [49].

To thoroughly explore actual reductions in the risk of OSAS, it would be intriguing to numerically quantify the reduction in incidence. This could be achieved by comparing subjects treated with MARPE, with SARPE and those who received no treatment. Additionally, understanding whether the changes reported are comparable to the results obtained with SARPE would provide valuable insight. This comparison could pave the way for proposing a treatment method that is significantly less invasive.

The most important limitation of this scoping review is the small sample size of quantitative analysis of the upper airway after MAPRE. These results should be interpretated with caution. In addition, there were differences in protocol expansion, patient age and settings of CBCT, and there were no medium and long-term follow-ups.

It could also be desirable to establish a protocol for a simple, reproducible evaluation method with a cost/benefit ratio. Such a method could be used routinely by clinicians, filling a current gap in the available options.

It is recommended to carry out more large sample trials in the future and obtain more precise and objective data to provide higher and more precise scientific quality.

## 5. Conclusions

MARPE treatment in adult patients aged 18 years and above results in statistically significant volumetric increases in the upper airways. Notably, the nasal cavities and nasopharynx exhibit the most substantial volumetric variations before and after treatment, with these areas being the primary focus for existing literature. However, there is a need for further studies to explore the remaining areas of the upper airway.

Considering the lack of homogeneity among the studies in the existing literature and the different methods employed for evaluating the upper airways, it is imperative to approach these results with caution. Furthermore, due to the objective anatomical complexity characterizing these structures, definitive conclusions are challenging.

An additional limitation is the uncertainty regarding the persistence of these changes over time. The majority of the selected studies did not include remote controls, preventing a comprehensive understanding of the long-term effects of miniscrew-assisted rapid palatal expansion (MARPE) treatment.

## Figures and Tables

**Table 1:** Data from the eight articles included in this review.

Authors and Year	Study Design	Participants	Expansion Device	Expansion Protocol	Measurement Method	Software	Airway Regions	Outcomes	Change Percentage %	Follow Up Point
Garcez et al., 2019 [27]	Case report	1 pt male Age 18	MSE	Twice a day (0.25 mm) until necessary expansion was achieved	CBCT volume Respiratory test	ITK-SNAP	Nasal cavity Pharyngeal airway	Increases in both nasal cavity and oropharyngeal volume	31%	T0: before expansion T1: immediately after expansion
Kim et al., 2018 [28]	Retrospective clinical study	14 pts (10 f, 4 m) mean age: 22.7 years range: 18.3–26.5 years	Four banded Hyrax MRE supported by four miniscrew	Once a day (0.2 mm/turn) until the required expansion was achieved	CBCT volume	On Demand 3d	Nasal cavity Nasopharynx	Volume and cross-sectional area of nasal cavity increased after MARME and were maintained after one year	NC-V 9.9% (T0–T1) 5.5% (T1–T2)—15.4% (T0–T2) NF 6.4% (T0–T1)—4.1% (T1–T2) 10.5% (T0–T2)	T0: before expansion T1: immediately after expansion T2: after one year exapnsion
Tang et al., 2021 [26]	Retrospective clinical study	30 pts (21 f, 9 m) mean age: 23.8 ± 3.90 years; range: 18–33 years	MSE type II	Once a day (0.13 mm/turn) until the required expansion was achieved	CBCT volume Computational fluid dynamics	Dolphin Images	Total Pharynx Nasopharynx Oropharynx Hypopharynx	Enlargements of the volume of total pharynx, nasopharynx and oropharynx were found	Pharynx 9.9% Nasopharynx 20.7% Oropharynx 8.84%	T0: before expansion T1: after 3 months
Li et al., 2020 [25]	Retrospective clinical study	22 pts (18 f, 4 m) mean age: 22.6 ± 4.5 years; range: 18–35 years	MSE	Twice a day (0.25 mm) until necessary expansion was achieved	CBCT volume	Dolphin Images	Nasal cavity Nasopharynx Retropalatal Retroglossal Hypopharynx	Volume of Nasal cavity and Nasopharynx increased significantly	V-NC 16.2% V-NPA 14.1% V-RPA 5.7% V-RGA 11.26 V-HPA −11.6%	T0: before expansion T1: immediately after expansion
Hur et al., 2017 [24]	Case report	1 pt male Age 18.7	Not reported	Not reported	CBCT volume and areas Computational fluid dynamics	ICEM-CFD	Nasal cavity Pharynx	The cross-sectional areas at most planes in nasal cavity and the upper half of the pharynx were significantly increased	N/A	T0: before expansion T1: after 6 months
Aneris et al., 2023 [29]	Controlled clinical trail	20 pts (man-to-woman ratio of 1:5,) mean age: 24.5 ± 6.2 years; range: 18–30 years	Hybrid with four miniscrew—Pec Lab, Bio Horizonte	Not reported	CBCT volume	Osirix MD	Total upper Retropalatal Retroglossal	Increases of all volumetric parameters and minimal transverse airway constriction (*p* < 0.05)	14%	T0: before expansion T1: after 120 days
Shetty et al., 2022 [23]	Retrospective clinical study	10 pts Range: 18–30 years	Not reported	Not reported	CBCT volume and linear measurements	Planmeca Romexis	Retropalatal Retroglossal Total airway	Slight decreases of retropalatal and retroglossal airway. All variations was found to be statististically insignificant	N/A	T0: before expansion T1: immediately after expansion

## Data Availability

No new data were created in this study. Data sharing is not applicable for this study.
